# Delayed Diagnosis Salicylate Toxicity With Confirmed Cerebrospinal Fluid Salicylate Concentration: A Case Report of Altered Mental Status Without Neuroglycopenia and With Coagulopathy

**DOI:** 10.7759/cureus.89613

**Published:** 2025-08-08

**Authors:** Mallika Singh, Joshua Bloom, Mary Ann Howland, Rana Biary, Mark Su

**Affiliations:** 1 Emergency Medicine, Jamaica Hospital Medical Center, Richmond Hills, USA; 2 Health and Mental Hygiene, New York City Poison Control Center, New York, USA; 3 Ronald O. Perelman Department of Emergency Medicine, Division of Medical Toxicology, New York University Grossman School of Medicine, New York, USA; 4 Emergency Medicine, Division of Medical Toxicology, New York City Health + Hospitals Jacobi/North Central Bronx, New York, USA; 5 Pharmacy Practice, College of Pharmacy and Health Sciences, St. John’s University, Queens, USA

**Keywords:** acquired coagulopathy, aspirin overdose, cerebrospinal fluid studies, intentional overdose, salicylate toxicity

## Abstract

Salicylate toxicity usually occurs as a result of elevated serum salicylate concentrations. Salicylate concentrations can be measured in cerebrospinal fluid (CSF), but the interpretation of these values is less well understood. Two phenomena believed to be associated with salicylate toxicity are neuroglycopenia and salicylate-induced coagulopathy, but these cases are typically not well-characterized. We report a case of delayed diagnosis of salicylate toxicity that was complicated by coagulopathy and had a quantifiable CSF salicylate concentration.

An 18-year-old female patient presented with abdominal pain and an altered mental state; she had previously presented at another hospital one day prior. She did not initially report salicylate overdose. A lumbar puncture was performed 4.5 hours after emergency department (ED) presentation to rule out meningitis. Due to additional history, a serum salicylate concentration obtained at 25 hours led to a result of 48.8 mg/dL (therapeutic range: 15-30 mg/dL). Hemodialysis (HD) was performed 37 hours after ED presentation due to the altered mental state. Prior to HD, the patient had a prothrombin time of 63 seconds (reference range: 11-13 seconds), international normalized ratio of 5.52 (reference range: 0.8-1.1), and activated partial thromboplastin time of 40.2 seconds (reference range: 25-35 seconds). After the case conclusion, waste CSF was tested and the CSF salicylate concentration was 23 mg/dL; the corresponding CSF and serum glucose concentrations were 60 mg/dL (reference range: 50-75 mg/dL) and 87 mg/dL (reference range: 70-99 mg/dL), respectively.

This is a case of delayed diagnosis salicylate poisoning complicated by coagulopathy, without neuroglycopenia. Although we do not recommend routine CSF testing in salicylate toxicity cases, analysis of CSF could shed insight into salicylate-induced encephalopathy. This case illustrates that prompt recognition and treatment of toxicity is key, as delayed diagnosis can increase morbidity and mortality.

## Introduction

Salicylate toxicity is commonly encountered due to its frequent presence in nonprescription medications [1,2]. While patient outcomes from acute salicylate toxicity are generally good, patients with a missed or delayed diagnosis of salicylate toxicity suffer from increased mortality [3]. Salicylate concentrations are typically measured in serum for diagnostic purposes and rarely obtained in other bodily fluids such as cerebrospinal fluid (CSF) [1,4].

Animal studies have demonstrated a correlation between salicylate serum and CSF concentrations and suggest that CSF salicylate concentrations could be more accurate than serum salicylate concentrations as a predictor of mortality [4-6]. Additionally, neuroglycopenia, also known as hypoglycorrhachia, has been noted to occur in salicylate-poisoned animals [1,2,7]. Although infrequently documented in humans, neuroglycopenia is believed to be a cause of altered mental status in salicylate-poisoned humans [1,5,8]. Multiple other abnormalities can occur as a complication of salicylate toxicity, including salicylate-induced coagulopathy. Although this phenomenon remains poorly described, the coagulopathy that occurs from salicylate is believed to be due to inhibition of platelet aggregation and salicylate effects on coagulation factors 2, 7, 9, and 10 [1].

Our objective was to report a case of a patient with delayed diagnosis of salicylate toxicity that was complicated by coagulopathy and who had a quantifiable CSF salicylate concentration without the presence of neuroglycopenia.

## Case presentation

An 18-year-old healthy female patient presented to an emergency department (ED) with abdominal pain and an altered mental state. According to the patient’s history (which could not be verified) she was seen at a different hospital for the same symptoms one day prior with reportedly normal laboratory tests and an unremarkable CT imaging of an unspecified body part(s). The patient did not report whether serum salicylate concentrations were obtained. Her abdominal pain had worsened since her prior visit. On presentation to the second ED, her vital signs were: blood pressure 114/60 mmHg; heart rate 70 beats/minute; respiratory rate, 18 breaths/minute; temperature 98.6°F; and O_2_ saturation 100% (room air). Her physical examination was notable for lethargy and diffuse abdominal tenderness. In the ED, she developed a fever of 100.4°F and altered mental status, prompting a lumbar puncture 4.5 hours after her presentation to the ED to rule out meningitis. Select laboratory analysis including blood, urine, and CSF studies are included (Tables 1-4).

**Table 1:** Laboratory values for basic metabolic panel, complete blood count, and hepatic panel throughout hospitalization ED: Emergency department

Time from ED Arrival	Reference Range	0:35	0:50	13:49	61:35
Basic Metabolic Panel					
Sodium	136-145 mEq/L	134	-	-	-
Potassium	3.5-5.0 mEq/L	3.9	-	-	-
Chloride	98-106 mEq/L	101	-	-	-
Bicarbonate	23-28 mEq/L	14	-	-	-
Blood Urea Nitrogen	8-20 mg/dL	17	-	-	-
Creatinine	0.50-1.10 mg/dL	0.9	-	-	-
Glucose	70-99 mg/dL	87	-	-	-
Calcium	8.6-10.2 mg/dL	7.7	-	-	-
Anion Gap	4-12 mmol/L	19	-	-	-
Complete Blood Count					
White Blood Cells	4,500-11,000/mm^3^	-	23	14.1	5.9
Hemoglobin	12-16 g/dL	-	11.7	9.8	8.2
Hematocrit	37%-47%	-	34.9	29.7	24.8
Platelet Count	150,000-450,000/μL	-	441	371	237
Hepatic Panel					
Total Bilirubin	0.3-1.0 mg/dL	0.4	-	0.4	0.5
Aspartate Transaminase	10-40 U/L	23	-	17	24
Alanine Transaminase	10-40 U/L	20	-	15	14
Alkaline Phosphatase	30-120 U/L	82	-	65	39
Total Protein	5.5-9.0 g/dL	7.4	-	5.7	4.8
Albumin	3.5-5.5 g/dL	4.2	-	3.2	2.8

**Table 2:** Laboratory values for CSF analysis throughout hospitalization ED: Emergency department; CSF: Cerebrospinal fluid; WBC: White blood cell; RBC: Red blood cell

Time from ED Arrival	Reference Range	4:35
CSF Appearance	Clear - Colorless	Clear
CSF Spun Appearance	Clear - Colorless	Clear
CSF WBCs	0-5 WBCs/mm^3^	0
CSF RBCs	0-5 RBCs/mm^3^	1
CSF Glucose	50-75 mg/dL	60
CSF Total Protein	15-45 mg/dL	<10

**Table 3:** Laboratory values for arterial blood gas throughout hospitalization ED: Emergency department

Time from ED Arrival	Reference Range	8:35	27:59	29:20	33:34	37:55	44:45	49:35	61:35
pH	7.35-5.45	7.392	7.354	7.382	7.472	7.592	7.601	-	7.437
paCO2	35-45 mmHg	23.1	25.7	25.9	22.7	30.4	35.3	-	48.5
paO2	80-100 mmHg	114	80.6	121	179	113	203	-	123
HCO3	22-26 mEq/L	17	16.5	17.5	-	31.5	-	-	31.4
O2 Saturation	95-100%	99.3	96.5	99.5	>100	100	>100.0	-	99.4
Base Excess	-2-+2 mEq/L	-10.9	-11.2	-9.7	-7.1	8.1	13.2	8.5	-

**Table 4:** Laboratory values for urine analysis and salicylate levels throughout hospitalization ED: Emergency department

Time from ED Arrival	Reference Range	1:45	4:35	25:00	25:15	28:05	33:14	38:17	40:55	44:29	49:35	50:10	56:35
Urine Analysis													
pH	4.5-8.0	5.5	-	-	5.5	-	-	6.5	-	-	>9.0	-	-
Salicylate Levels													
CSF Salicylate	<0.5 mg/dL	-	23	-	-	-	-	-	-	-	-	-	-
Serum Salicylate	<1 mg/dL	-	-	48.8	-	46.8	44.9	40.7	9.6	6.2	-	2.5	<1

During her second ED visit, the patient underwent a transvaginal ultrasound two hours after ED presentation for abdominal pain that visualized a right ovarian cyst. She also had an unremarkable CT brain scan that was ordered due to altered mental status. She underwent a CT scan of the abdomen and pelvis, which noted radio-opaque foreign bodies in the colon that were deemed to be consistent with “pill fragments” according to the consulting gastroenterology service. On re-interview, the patient confirmed taking an unknown quantity of aspirin pills approximately 36 hours before arrival to the second hospital. A serum salicylate concentration obtained at 25 hours after the second ED presentation resulted in a value of 48.8 mg/dL (therapeutic range: 15-30 mg/dL). The patient's urine drug screen resulted negative for all substances tested.

**Figure 1:**
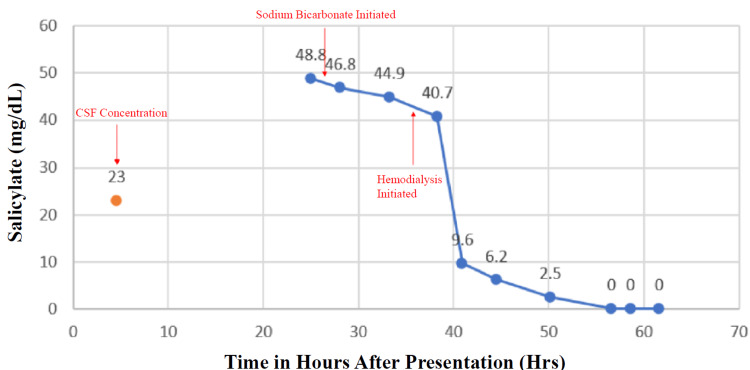
Measured salicylate concentration throughout hospitalization. Time 0 listed as presentation to the ED. ED: Emergency department

The regional poison center was consulted and recommended treatment with IV sodium bicarbonate, and the patient’s urine and serum were adequately alkalinized (urine pH > 8). Hemodialysis (HD) was also recommended due to a concern for salicylate-induced encephalopathy. The patient’s serum salicylate concentration decreased with sodium bicarbonate infusion and HD (Figure 1).

**Figure 2:**
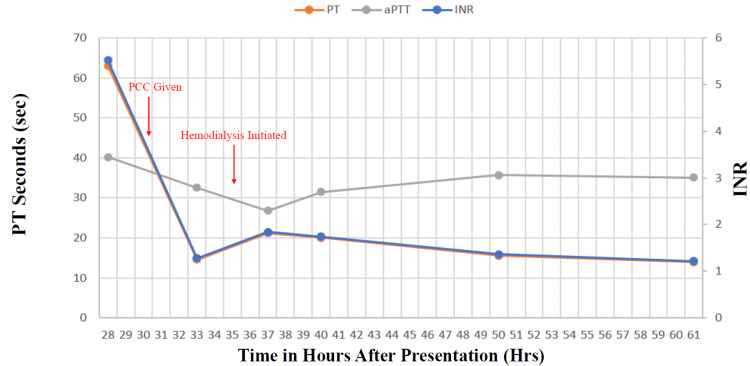
Coagulation studies throughout hospitalization Laboratory reference ranges: International normalized ratio: 0.85-1.18; Prothrombin time: 9.5-13.0 seconds; Activated prothrombin time: 24.5-35.6 seconds PCC: Prothrombin complex concentration

Prior to the placement of the HD catheter, the patient’s coagulation studies were abnormal, despite normal hepatic function and lack of reported use of oral anticoagulants, vitamin K antagonists, or heparin, raising concern for salicylate-induced coagulopathy. Prothrombin time was 63 seconds (reference range: 11-13 seconds), international normalized ratio was 5.52 (reference range: 0.8-1.1), and activated partial thromboplastin time was 40.2 seconds (reference range: 25-35 seconds). The patient was administered 2,500 units of prothrombin complex concentrate (PCC) before catheter placement, which corrected the coagulopathy (Figure 2).

On hospital day 4, the patient’s abdominal pain and encephalopathy resolved. She was medically cleared with no noted deficits and transferred to the inpatient psychiatry service.

After the conclusion of this case, waste CSF from the patient’s lumbar puncture from hospital day 1 was tested for salicylate concentration. The CSF salicylate concentration was 23 mg/dL with a CSF glucose concentration of 60 mg/dL (reference range: 50-75 mg/dL); the corresponding serum glucose concentration drawn three hours before this CSF was obtained was 87 mg/dL (reference range: 70-99 mg/dL).

## Discussion

Acetylsalicylate is absorbed in the stomach and small intestines after ingestion. As the dose increases from therapeutic to overdose, elimination changes from a first-order process to zero-order. Despite salicylates undergoing zero-order kinetics, attempting to calculate prior salicylate concentrations in late presentations may be inaccurate due to the formation of salicylate bezoars and altered gastrointestinal motility, as seen with the presence of pill fragments on the patient’s CT scan. As salicylate concentrations rise, the salicylate exceeds the limit of protein binding in the serum. As the environment becomes more acidic, the volume of distribution of salicylate increases, which leads to increased CSF penetration and neurotoxicity. The cause of death in salicylism is often attributed to seizures and cerebral edema leading to neuronal injury [4].

Animal studies have shown a correlation between serum and CSF concentrations. Our case shows a lower CSF salicylate concentration, 23 mg/dL, than the proposed lethal concentration of 35-55 mg/dL observed in animal studies [4,9]. However, there were no human studies of lethal versus symptomatic CSF concentrations discovered upon literature review.

Discrepancies between the higher CSF salicylate concentrations found in animal studies and the low CSF salicylate concentration seen in our patient may be due to the patient not having a salicylate concentration consistent with lethality. The patient’s CSF salicylate concentration of 23 mg/dL is still elevated and could have contributed to the patient’s altered mental status.

Additionally, animal studies have documented that salicylate-poisoned subjects suffer from neuroglycopenia. CSF glucose concentrations are reduced compared to non-poisoned subjects despite normal serum glucose concentration [2,5,7]. In our case, the patient’s serum glucose of 87 mg/dL and CSF glucose of 67 mg/dL, was not consistent with neuroglycopenia. The lack of neuroglycopenia in our patient may be related to her relatively low CSF salicylate concentration.

The coagulopathy caused by salicylate poisoning is believed to be secondary to irreversible acetylation of cyclooxygenase-1 and cyclooxygenase-2, preventing thromboxane A2 formation and inhibiting platelet aggregation. Additionally, salicylate causes a dose-dependent decrease of gamma-carboxyglutamate-containing coagulation factor concentrations (factors 2, 7, 9, and 10), acting similarly to warfarin; this leads to the prolongation of prothrombin time and international normalized ratio. However, there are only a few reports of clinical coagulopathy or major bleeding from salicylate toxicity [3,10]. Since bleeding is rare and coagulation studies are not routinely investigated in salicylism, the prevalence of salicylate-induced coagulopathy is unknown. Laboratory testing was consistent with a salicylate-induced coagulopathy, without reported bleeding events. The coagulopathy was corrected with the administration of PCC and concomitant treatment of the patient’s salicylate toxicity. Furthermore, the patient was not taking any oral anticoagulants.

Limitations

In this case of salicylate toxicity, several limitations exist. The absence of paired serum and CSF salicylate concentrations, as well as the lack of early serum salicylate measurements during hospital presentation, hinder the ability to fully assess the toxicokinetic profile. The patient’s low CSF salicylate concentration could suggest she did not have salicylism; however, due to her blood work reveling a mixed acid-base disorder and her altered mental status that improved with HD, it is likely the patient had late-presenting signs of salicylate toxicity. Additionally, respiratory rates are frequently documented incorrectly in clinical settings, which may further obscure the clinical picture of possible tachypnea in salicylate toxicity cases [11]. Although the respiratory rate was documented as 18, other findings, such as a base deficit of -10 and partial pressure of CO_2_ (PCO_2_) of 23.1 mmHg, were more indicative of the mixed respiratory acidosis typically seen in late-presenting salicylate toxicity, casting doubt on the accuracy of the recorded respiratory rate. It is possible that the patient’s coagulation studies were affected by collection errors such as underfill, and the patient’s minimal partial thromboplastin time prolongation could correspond to this; however, her PT and INR were prolonged and consistent across multiple draws; this has previously been reported in salicylism. The resolution of the patient's coagulopathy may be secondary to salicylate clearance, however, also may be due to PCC administration [12]. Additionally, our CSF analysis does not address other proposed mechanisms of neurotoxicity in salicylism, such as neuronal and glial mitochondrial toxicity [13].

## Conclusions

Prompt recognition and treatment of toxicity is key, as delayed diagnosis can increase morbidity and mortality. This case illustrates that vigilance is required to make the diagnosis of salicylate toxicity in the absence of supportive history. This case did not demonstrate neuroglycopenia; however, it did demonstrate salicylate-induced coagulopathy. While performing CSF analysis routinely for salicylate measurement is unnecessary, if excess CSF were to be available from patients who are found to have salicylate toxicity, further studies could aid in understanding the changes in CSF due to salicylate toxicity could elucidate the neurologic effects of salicylate poisoning.
